# Temperature and humidity-dependent interaction effects on tongue color in diabetic patients: a quantitative analysis and TCM perspective

**DOI:** 10.3389/fendo.2026.1772207

**Published:** 2026-03-25

**Authors:** Bingnan Zhang, Naijin Zhang, Huixia Ren, Yonghui Li, Haiyan Du, Tingjing Ren, Jiayu Huang, Hongwu Wang

**Affiliations:** School of Public Health and Health Sciences, Tianjin University of Traditional Chinese Medicine, Tianjin, China

**Keywords:** cardiovascular complications, diabetes, objective tongue image, relative humidity, temperature, tongue diagnosis

## Abstract

**Objective:**

To explore the effects of temperature and relative humidity on the tongue color of diabetic patients with different severity levels and their interactions.

**Methods:**

A total of 1,711 participants were included, divided into five groups based on disease status (no diabetes, simple diabetes, diabetes with hypertension, diabetes with coronary heart disease, diabetes with both hypertension and coronary heart disease). RGB values of the tongue body and coating at five sites (tip, middle, root, left, and right) were collected (30 indicators in total). The month of visit (six categories) was used as a composite temperature-humidity proxy variable, with age, gender, BMI, smoking, and alcohol consumption as covariates. Multiple linear regression was conducted, including the interaction term of month × group, to perform Type III tests and FDR corrections for the 30 RGB indicators and principal component scores.

**Results:**

Significant interaction effects were found between the month of visit and disease group on tongue color (PC1: F(20,1670)=12.87, P<0.0001; PC2: F(20,1670)=18.64, P<0.0001). In the diabetes with hypertension and coronary heart disease group, tongue redness and brightness increased by 2.74–3.19 standard deviations during the hot and humid months of July and August compared to colder months (P<0.0001), while the no diabetes group remained stable throughout the year. Forty-seven RGB interaction terms had FDR <0.05, with the ten most significant located at the tongue tip; the R-value of the tongue body was significantly associated with temperature increase, showing a clear dose-response relationship with the severity of complications (trend P<10^-^¹²).

**Conclusion:**

High temperature and humidity can significantly amplify tongue redness in diabetic patients, potentially involving a microcirculation expansion-endothelial dysfunction-oxidative stress cascade pathway, while tongue color remains highly stable in healthy individuals. This interaction effect suggests that RGB tongue indicators may serve as an objective dynamic biomarker reflecting cardiovascular metabolic risk under heat stress.

## 1 Introduction

Diabetes and its cardiovascular complications have emerged as a significant global chronic disease burden (1). Traditional Chinese Medicine (TCM) tongue diagnosis, a non-invasive, holistic, and dynamic method, offers distinct advantages in syndrome differentiation and disease assessment for diabetes and its complications (2). TCM posits that “the tongue is the sprout of the heart” and that “the spleen and stomach are the foundation of postnatal health, responding externally through the lips and tongue.” (3) The tongue image is thought to reflect the interaction between the internal organs’ qi and blood, as well as the external influence of seasonal pathogens (4). The “Su Wen: Zhi Zhen Yao Da Lun” states: “All swelling and fullness are related to the spleen” and “excessive heat causes the blood color to be externally visible,” suggesting that high temperature and humidity environments may exacerbate stagnation of qi and blood, as well as the manifestation of heat in diabetic patients (5). However, previous studies on the objectification of tongue diagnosis have primarily focused on cross-sectional comparisons of disease states, with limited attention given to the seasonal fluctuations of climatic factors, particularly temperature and humidity, on tongue color (6). Furthermore, there has been a lack of systematic investigation into the interaction between climate and disease state (7). Modern research indicates that high temperature and humidity can trigger sympathetic nervous system excitation, peripheral microcirculation expansion, increased oxidative stress, and subsequent endothelial dysfunction (8). Diabetic patients with hypertension and coronary heart disease already experience vascular endothelial dysfunction, microvascular lesions, and chronic low-grade inflammation, which significantly diminish their compensatory ability to heat stress (9). Theoretically, this may result in abnormal amplification of blood flow to the tongue mucosa and an increase in oxygenated hemoglobin content, which would manifest as significant seasonal changes in tongue redness and brightness (10). In contrast, healthy individuals, with intact endothelial reserves and antioxidant systems, may maintain relatively stable tongue color (11).

If the existence of this interaction effect and its dose-response relationship with disease severity can be confirmed, it may offer a novel climate-disease interaction dimension for the objectification of tongue diagnosis (12), potentially serving as a biomarker for early detection of cardiovascular complications in diabetic patients during hot seasons (13). This study involved 1,711 patients with varying degrees of diabetes and cardiovascular complications (14). By employing standardized RGB quantitative analysis of tongue images (15), combined with principal component dimensionality reduction (16) and rigorous interaction statistical modeling (17), this study systematically investigates the main effects of the visit month (a composite proxy for temperature and relative humidity) on tongue color and its interaction with disease groups (18). The goal is to elucidate the modifying effect of climate factors on the objective expression of diabetes tongue images, providing modern scientific evidence for the TCM theory of “harmony between heaven and man.” (19).

## 2 Methods

### 2.1 Study population

A six-month cross-sectional study was conducted from June to November, 2023 at the Second Affiliated Hospital of Tianjin University of Traditional Chinese Medicine in Tianjin, China. The study participants were patients from the outpatient and inpatient departments of the Endocrinology Division. The inclusion criteria were as follows: aged 18 years or older (regardless of gender), conscious with normal cognitive function, able to fully understand the purpose and content of the study, and voluntarily sign an informed consent form; able to cooperate with the tongue image collection equipment and complete standardized, comprehensive, and high-quality tongue image capture; no consumption of food that significantly stains the tongue or medications that could alter tongue color within the past seven days; no acute diseases (e.g., respiratory infections, acute gastroenteritis) within the past 14 days that could cause significant tongue abnormalities; no malignancies, hematologic diseases, or other serious systemic conditions, and no mental disorders or severe cognitive impairments; able to independently and truthfully complete all questionnaires in accordance with the study protocol. After applying these criteria, a final cohort of 1,711 individuals was included. Participants were categorized into five groups based on the following standardized clinical criteria: (1) Diabetes was diagnosed according to the American Diabetes Association (ADA) 2023 guidelines (HbA1c ≥ 6.5% or fasting plasma glucose ≥ 126 mg/dL) (20); (2) Hypertension was defined as systolic blood pressure ≥ 140 mmHg and/or diastolic blood pressure ≥ 90 mmHg, or current use of antihypertensive medication (21); (3) Coronary heart disease was identified based on a history of myocardial infarction, coronary artery bypass grafting, or significant stenosis confirmed by coronary angiography. These standardized definitions were applied to all 1,711 participants to ensure consistent grouping and to confirm the absence of these conditions in the healthy control group.

The detailed inclusion and exclusion process is shown in Flowchart 1. All participants voluntarily filled out questionnaires and had their tongue images captured using the TFDA-1 device. The study received approval from the ethics review board of the research institution, and each participant signed an informed consent form before the formal examination (**Supplementary Material S1**). The study was formally approved by the Ethics Committee of Tianjin University of Traditional Chinese Medicine (Approval No.: TJUTCM-EC20190004).

### 2.2 Climate data

The “China Meteorological Yearbook,” a comprehensive reference published by the China Meteorological Administration, was consulted (22). It records the activities and progress of national meteorological departments in areas such as business, research, and education, along with nationwide weather and climate summaries and impact assessments (23). The yearbook also provides statistical data from various departments of the China Meteorological Administration (24, 25). Data collection spanned from May to October. In this study, the month of visit was utilized as a composite proxy variable for environmental heat stress. According to the *China Meteorological Yearbook*, these months in Tianjin represent a distinct gradient of monthly mean temperature and relative humidity. This approach captures the sustained seasonal environmental exposure which is physiologically relevant to the chronic microcirculatory status of diabetic patients (26).

### 2.3 Tongue image data collection

#### 2.3.1 Tongue diagnosis instrument

The TFDA-1 desktop tongue diagnosis instrument used in this study was independently developed and provided by the Smart Diagnostic Technology Research Laboratory of Shanghai University of Traditional Chinese Medicine (**Supplementary Material S3**). This device is a specialized medical tool designed to objectify and standardize TCM tongue diagnosis. It uses a high-precision imaging system (digital camera or professional imaging sensor) to capture and digitally store tongue images in a closed, constant lighting environment, assisting in TCM clinical syndrome differentiation.

The instrument consists mainly of a constant light source, a light-blocking collection box, a high-definition imaging module, a jaw support frame, and a display screen. Its optical performance meets strict standards: illuminance range of 1,000–8,000 lx, color temperature of 4,500–7,000 K, color rendering index Ra > 85, spatial resolution ≥ 5 lp/mm, color reproduction color difference ΔE ≤ 20, and relative distortion ≤ ± 5%. The shooting parameters were set as follows: cool white LED as the main light source, illuminance of approximately 2350 lx, color temperature fixed at 5000 K, manual mode (M), shutter speed of 1/125 s, aperture f/6.3, and ISO sensitivity of 200. These settings ensured high-fidelity reproduction of key tongue features, such as color, coating, and shape, providing accurate and reproducible objective tongue diagnosis data for clinical use.

#### 2.3.2 Collection process

Following the standardized tongue image collection guidelines established by the National Key R&D Program, all researchers involved in the collection underwent systematic training to become proficient in the operation procedures and use of the TFDA-1 instrument. Before the collection, researchers explained the process to participants, alleviated any anxiety, and demonstrated the correct tongue stretching posture. Participants were then instructed to sit upright, place their chin steadily on the chin support of the instrument, close their eyes, and keep their head still.

When instructed, participants were asked to open their mouth quickly, relax, and naturally extend their tongue, ensuring it was fully extended with the tip hanging down naturally and the surface fully exposed. They were asked to hold the tongue stable for 3 seconds, after which the researcher quickly took the image and archived it with a code. Once captured, the image was immediately checked on the display screen for quality. If issues such as fog, light leakage, overexposure, or blurring were identified, the participant was given 1–2 minutes of rest before the image was retaken. After each collection, the chin support and other areas that came into contact with the participant were thoroughly wiped and disinfected with 75% alcohol to ensure hygiene. If the participant experienced dizziness, nausea, or oral discomfort during the collection, the procedure was immediately stopped, and the participant’s health was monitored.

Throughout the collection process, participants were instructed to remain relaxed, natural, and stable, while emphasizing image clarity, proper operation, and environmental and instrument hygiene to ensure the standardization and high reproducibility of all data.

All researchers underwent systematic training. Image collection followed the rigorous requirements established by the National Key R&D Program. Specifically, tongue images were captured in the morning before breakfast. Participants were instructed to avoid strenuous activity and food/drugs that might stain the tongue for at least 30 minutes prior to collection. A 3-to-5-minute rest after mouth rinsing was mandatory if food residues were present. During capture, participants lean slightly forward with their jaw on the support, naturally extending 1/2 to 2/3 of the tongue surface [New Ref X]. The focus was manually set on the central region of the tongue body via the touch-screen interface to ensure optimal exposure and clarity. After shooting, the image quality was immediately verified. Any images exhibiting overexposure, insufficient light, or fog occlusion were immediately excluded from the analysis.

#### 2.3.3 Tongue region division

For color analysis, the tongue diagnosis instrument automatically divides the captured image into five functional areas based on the traditional TCM five-section theory, as illustrated in **Supplementary Figure 2**.The tongue is divided longitudinally into the tip (1/5), middle (3/5), and root (1/5). Transversely, the tongue is divided by the midline, extending about 1/5 of the width to each side, resulting in the following five characteristic regions:

Tip Region: Located at the central part of the front 1/5 of the tongue, primarily corresponding to the heart.

Left Region: Located at the lower middle part of the left 3/5 of the tongue, mainly reflecting the left spleen-stomach or liver-gallbladder.

Right Region: Located at the front middle part of the right 3/5 of the tongue, mainly reflecting the right lung or liver-gallbladder.

Middle Region: Located in the central rectangular area of the middle 3/5 of the tongue (marked with a red dot in the diagram), reflecting the overall function of the spleen-stomach.

Root Region: Located in the upper triangular area and the posterior sides of the last 1/5 of the tongue, primarily corresponding to the kidneys and lower abdomen.

This division allows for accurate analysis of tongue image, coating, and shape in relation to different internal organ projections on the tongue, enabling regional and objective judgments of organ pathology.

### 2.4 Statistical analysis

#### 2.4.1 Comparison of baseline characteristics

For normally distributed continuous data, values are presented as ± s, and for skewed data, values are presented as M (Q1, Q3). Categorical data are presented as n (%)Comparisons of continuous data among multiple groups were performed using one-way analysis of variance (ANOVA) or the Kruskal-Wallis H test, depending on normality and homogeneity of variance. Pearson’s χ² test or Fisher’s exact test was used for categorical data.

#### 2.4.2 Main effects and interaction effects of visit month and disease group on tongue color (main analysis)

The outcome variables were 30 tongue RGB indicators (R, G, B values for thetongue color and coating at five sites: tip, middle, root, left, and right). Since each participant had a single tongue color collection and the temperature and relative humidity of the visit month were highly correlated, with uneven distribution across the five groups, the visit month (a six-category variable, with November as the reference) was used as a proxy for temperature-humidity. A multiple linear regression model was established for each RGB indicator, with fixed effects including: visit month (6 categories); disease group (5 categories, with group 1, no diabetes, as the reference); the visit month × disease group interaction term; covariates: age (continuous), gender (male/female), BMI (continuous), smoking (yes/no), alcohol consumption (yes/no).

Type III sum of squares was used for overall effect testing. All interaction term p-values for the 30 outcome variables were corrected using the Benjamini-Hochberg method for false discovery rate (FDR). *Post-hoc* pairwise comparisons and corrected predicted means and slope estimates were conducted using the emmeans package (Tukey method for multiple corrections). Key results were presented using corrected predicted trajectory plots, volcano plots (F value vs. -log_10_(FDR)), top 10 interaction effect ranking plots, and tongue site heatmaps.

#### 2.4.3 Principal component analysis

After standardizing the 30 RGB indicators using z-scores, principal component analysis (PCA, without rotation) was performed based on the correlation matrix. The first eight principal components had eigenvalues > 1, explaining 85.0% of the total variance, with PC1 explaining 63.1% (mainly reflecting overall brightness and redness) and PC2 explaining 19.2% (mainly reflecting red-green contrast). PC1 and PC2 accounted for 63.1% and 19.2% of the total variance, respectively. The primary objective of using PCA in this study was to address the multiple testing problem. By reducing the 30 inter-correlated tongue color indicators into a set of orthogonal (uncorrelated) principal components, we effectively minimized the redundancy of information and reduced the risk of Type I error (false positives). This dimensionality reduction strategy ensures that our analysis focuses on the most representative features of tongue manifestations while maintaining statistical rigor (27, 28).The eight principal component scores were then used as outcome variables, and the same model as described in section 2.4.2 was repeated to validate the robustness of the results. PCA and visualization were performed using the prcomp(), factoextra, and ggplot2 packages.

#### 2.4.4 Statistical software and packages

All statistical analyzes were performed using R 4.5.2 software with two-sided tests, with significance defined as p < 0.05 or FDR < 0.05. The primary R packages used were: stats, car, emmeans, multcomp, ggplot2, ggpubr, dplyr, tidyr, factoextra, ComplexHeatmap, RColorBrewer, and others. All analysis code was reproducible.

## 3 Discussion

This study identified significant, disease severity-dependent interaction effects of visit month (a composite proxy for temperature and relative humidity) on tongue color in diabetic patients (26). Specifically, diabetic patients with hypertension and coronary heart disease exhibited markedly increased tongue redness and brightness (PC1, PC2, and most original RGB indicators) during the high temperature and humidity season (July-August) compared to the cold season (29). In contrast, the tongue color of the no diabetes group remained stable throughout the year (30). The tongue tip was found to be the most sensitive region to this interaction effect (31). Furthermore, the R value of the tongue body in the middle region showed a clear dose-response relationship with temperature increases and the number of complications (32). These findings provide modern quantitative evidence for the Traditional Chinese Medicine (TCM) theory of “harmony between heaven and man.” (33) TCM asserts that “excessive heat causes the blood color to show externally, with the tongue becoming red and the coating yellow.” (34) However, previous research has generally been limited to cross-sectional observations in healthy populations or a single season, making it difficult to distinguish between the primary effects of climate and the modifying effects of disease status (35). This study, with its large sample size (1,711 participants), multiple outcomes (30 RGB indicators), rigorous control for confounders, and explicit testing of interaction terms, is the first to confirm that only the most severe subgroup of diabetes with hypertension and coronary heart disease experiences significant amplification of tongue redness under high temperature and humidity (36). In contrast, healthy individuals maintain a strong homeostasis of tongue color (37). This suggests that tongue “redness” may serve as a dynamic biomarker reflecting cardiovascular metabolic stress in diabetic patients (38).

These results provide objective evidence supporting the TCM concepts of “harmony between heaven and man” and “excessive heat harms qi, and when heat is intense, blood color shows.” (39) This study suggests that tongue redness could act as a dynamic biomarker reflecting cardiovascular metabolic stress in diabetes (40). Its physiological basis may lie in the expansion of microcirculation and the amplification of oxidative stress induced by high temperature and humidity, particularly in severe diabetic patients with endothelial dysfunction, while healthy individuals can maintain a stable tongue color due to intact endothelial reserves and robust antioxidant systems (41). From a physiological perspective, high temperature and humidity induce significant increases in tongue mucosal microcirculation blood flow and oxygenated hemoglobin suggesting the potential for the following hypothetical cascade pathway (42):

**Core body temperature increase** activates the hypothalamus-sympathetic-adrenal medulla axis, releasing catecholamines that cause dilation of the tongue mucosal capillaries, increasing blood flow by 2–4 times (43).

**High environmental humidity** inhibits sweat evaporation, and the body compensates by further dilating skin-mucosal blood vessels to increase radiant heat dissipation. The tongue surface reacts most dramatically due to its high metabolic rate and rich blood vessel network (44). Diabetic patients with hypertension and coronary heart disease exhibit severe endothelial dysfunction (eNOS downregulation, ADMA accumulation, reduced NO bioavailability, and overexpression of ET-1), depleting the heat-induced vascular dilation reserve and causing pathological over-perfusion of local blood flow (45). Heat stress activates NADPH oxidase and uncoupled eNOS, generating superoxide anions (O_2_^-^), which rapidly react with NO to form peroxynitrite anions (ONOO^-^), further damaging the capillary endothelial barrier and causing mild erythrocyte extravasation and accumulation of oxygenated hemoglobin in the tongue body. This macroscopically manifests as a significant increase in the R value and brightness (46). Chronic hyperglycemia induces advanced glycation end products (AGEs) that bind to RAGE, upregulating the NF-κB pathway and exacerbating local inflammatory microenvironments. This leads to rapid increases in IL-6, TNF-α, and VEGF under heat stress, increasing the permeability of tongue mucosal capillaries and the fragility of newly formed microvessels (47).

This pathological amplification effect is counterbalanced in healthy individuals by intact endothelial reserves, efficient antioxidant systems (e.g., SOD, GPx), and high-efficiency heat shock proteins (HSP70/90), which help stabilize tongue color (48). In contrast, patients with the most severe vascular complications (Group 5) experience nearly complete loss of this compensation, leading to a significant increase in tongue redness, particularly at the tongue tip (the most richly supplied by blood and most sensitive to sympathetic innervation) (49). This finding strongly aligns with TCM’s description of the tongue as the “sprout of the heart” and the belief that “excessive heat causes blood to show externally.” (50) The strengths of this study include the large sample size, multiple outcome variables, dual validation of both raw and reduced-dimensional data, and rigorous testing of interaction terms with false discovery rate (FDR) correction (27). The limitations of the study lie primarily in its cross-sectional design and the inability to adjust for seasonal variations in medication doses for antihyperglycemic and antihypertensive drugs, as well as immediate dietary and exercise status (51). In conclusion, high temperature and humidity significantly amplify tongue redness in diabetic patients with cardiovascular complications which may be explained by a potential cascade mechanism involving sympathetic activation, endothelial dysfunction, oxidative stress, and microcirculatory over-perfusion (52). These findings suggest that RGB tongue image indicators may serve as objective, dynamic biomarkers reflecting cardiovascular metabolic risk under heat stress (53). Further prospective, multi-point follow-up studies are warranted to validate these results (54).

## Data Availability

The raw data supporting the conclusions of this article will be made available by the authors, without undue reservation.
