# Supplementary figures and images for: Temperature and humidity-dependent interaction effects on tongue color in diabetic patients: a quantitative analysis and TCM perspective
Source: Front Endocrinol (Lausanne). 2026 Mar 25;17:1772207. doi: 10.3389/fendo.2026.1772207 (PMC13056864; doi:10.3389/fendo.2026.1772207)

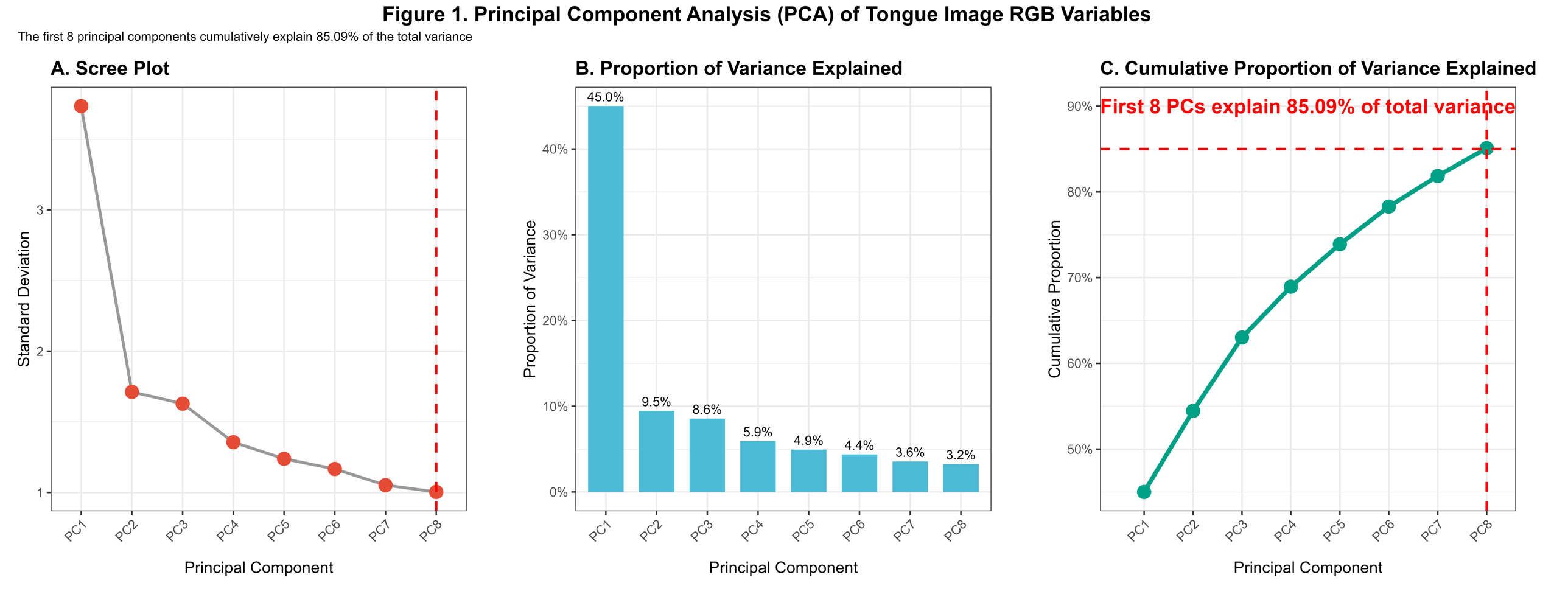

Supplement: Supplementary file 1 [file Image1.png]

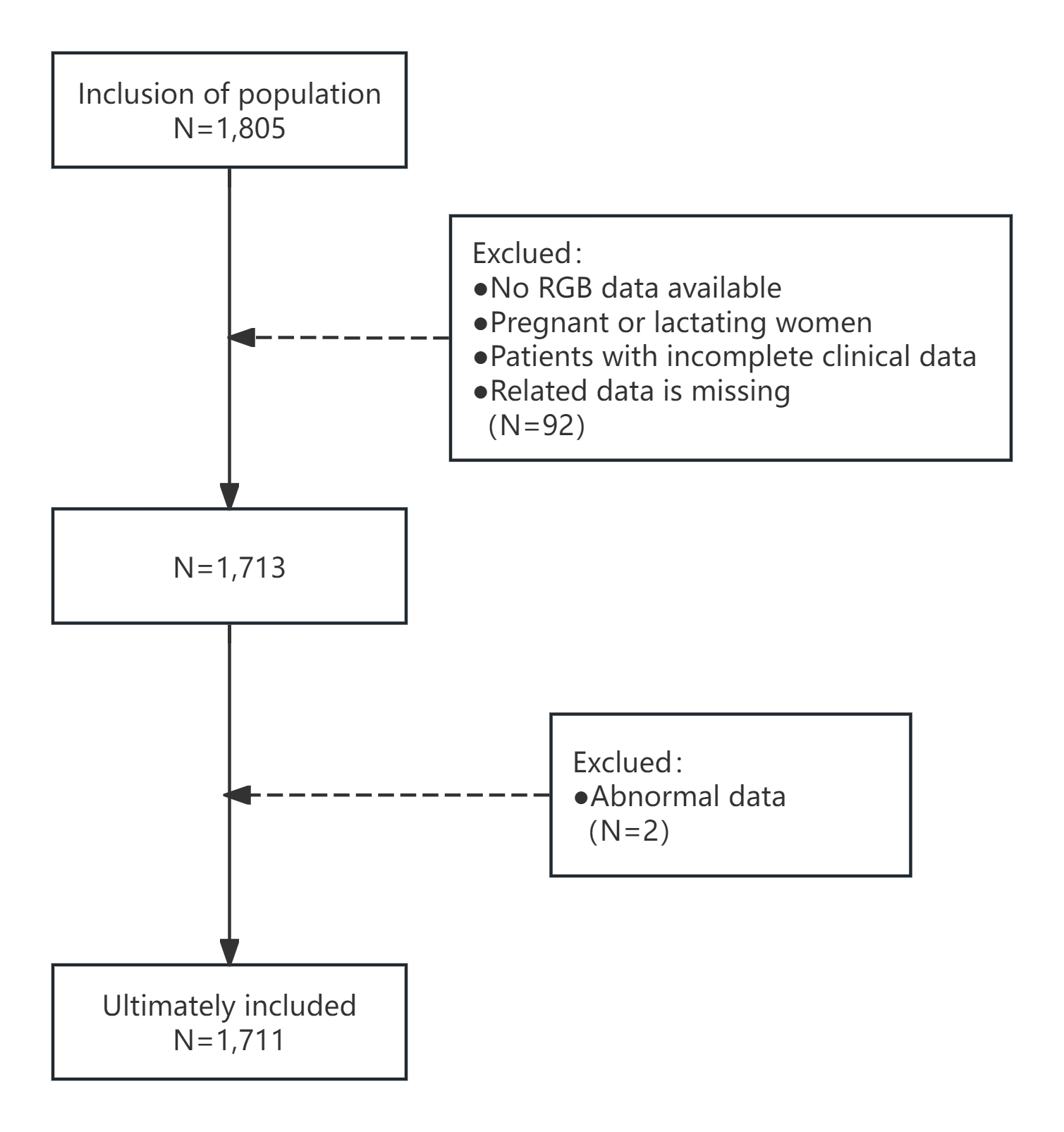

Supplement: Supplementary file 2 [file Image2.png]

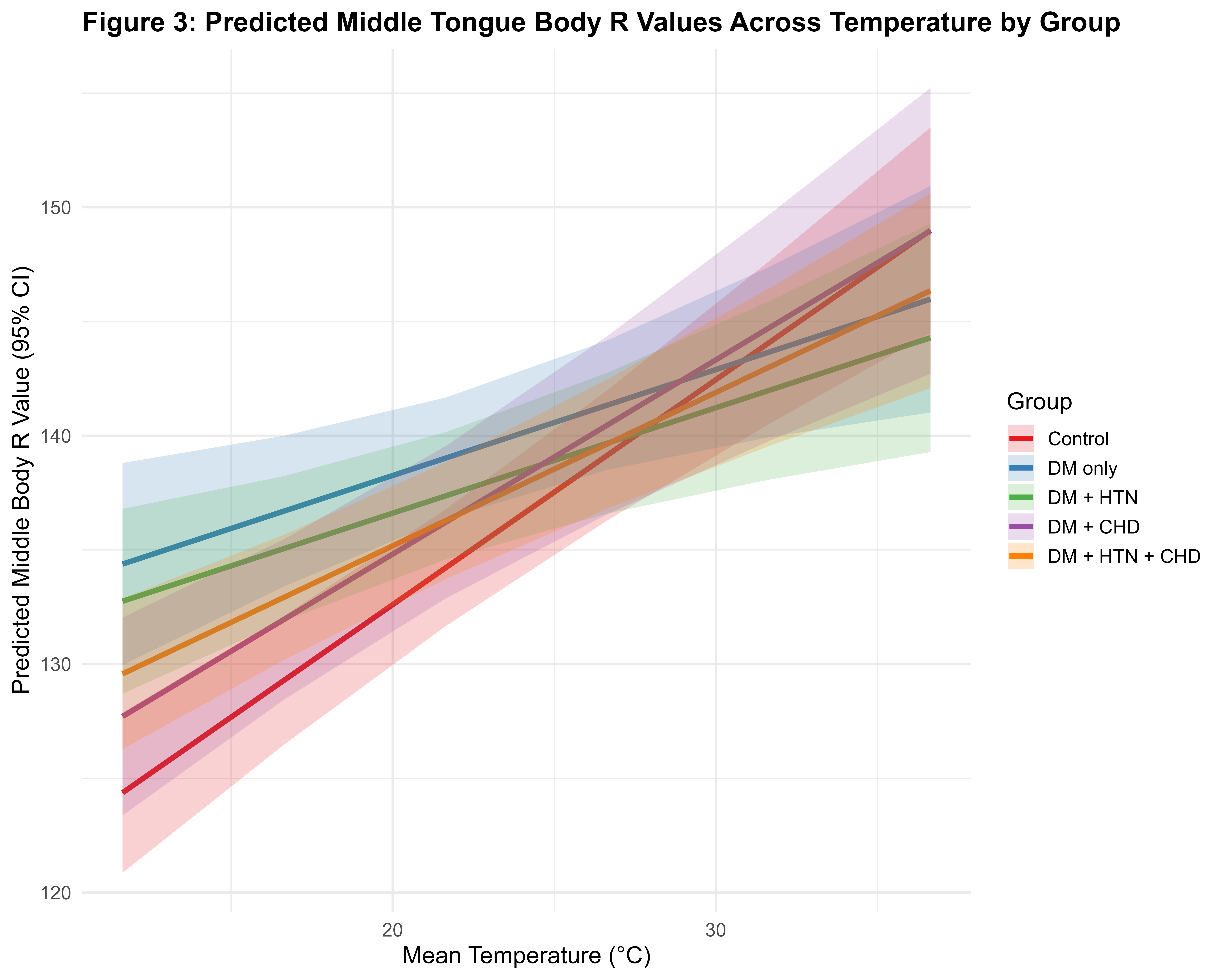

Supplement: Supplementary file 3 [file Image3.png]

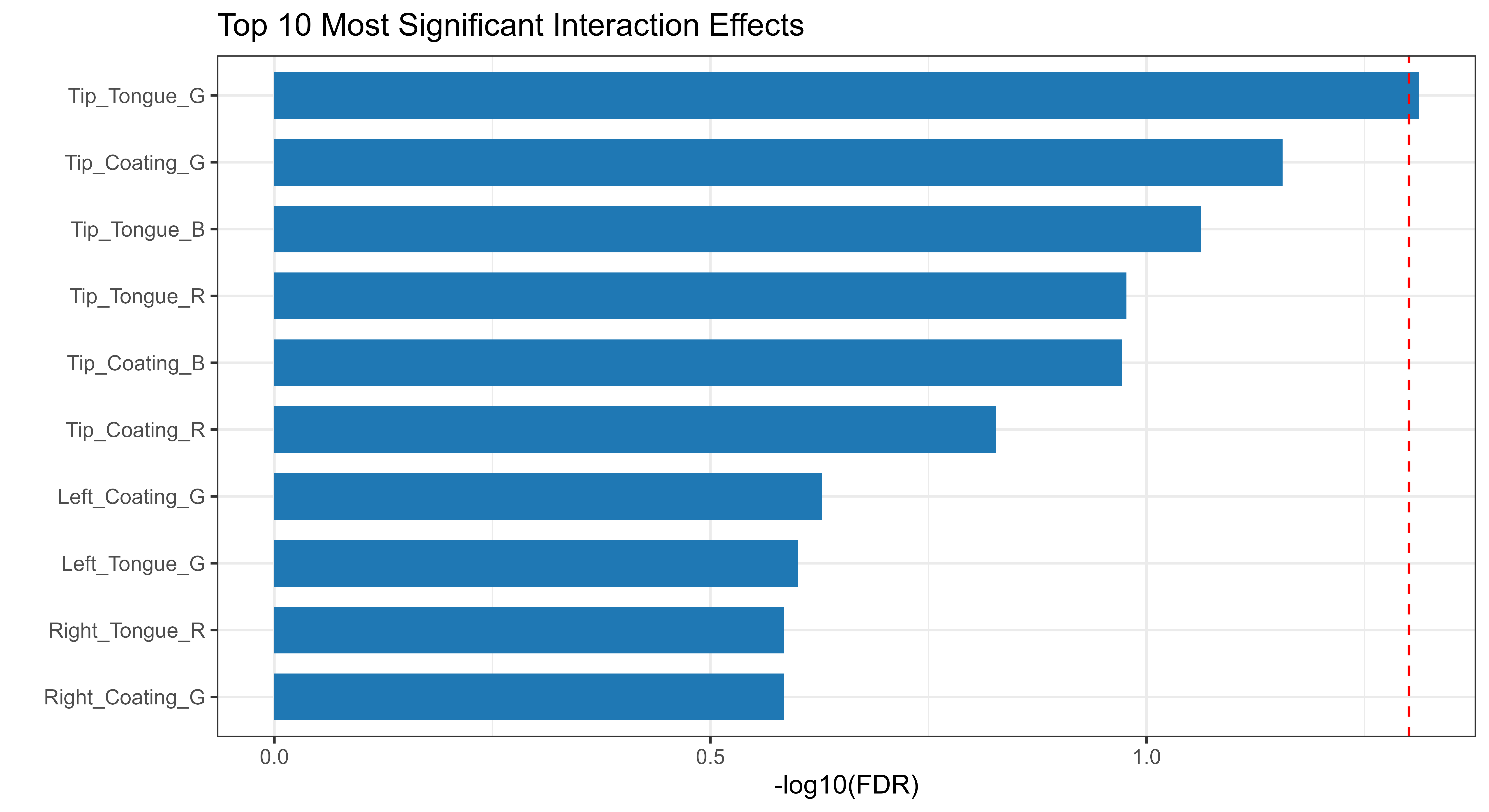

Supplement: Supplementary file 4 [file Image4.png]

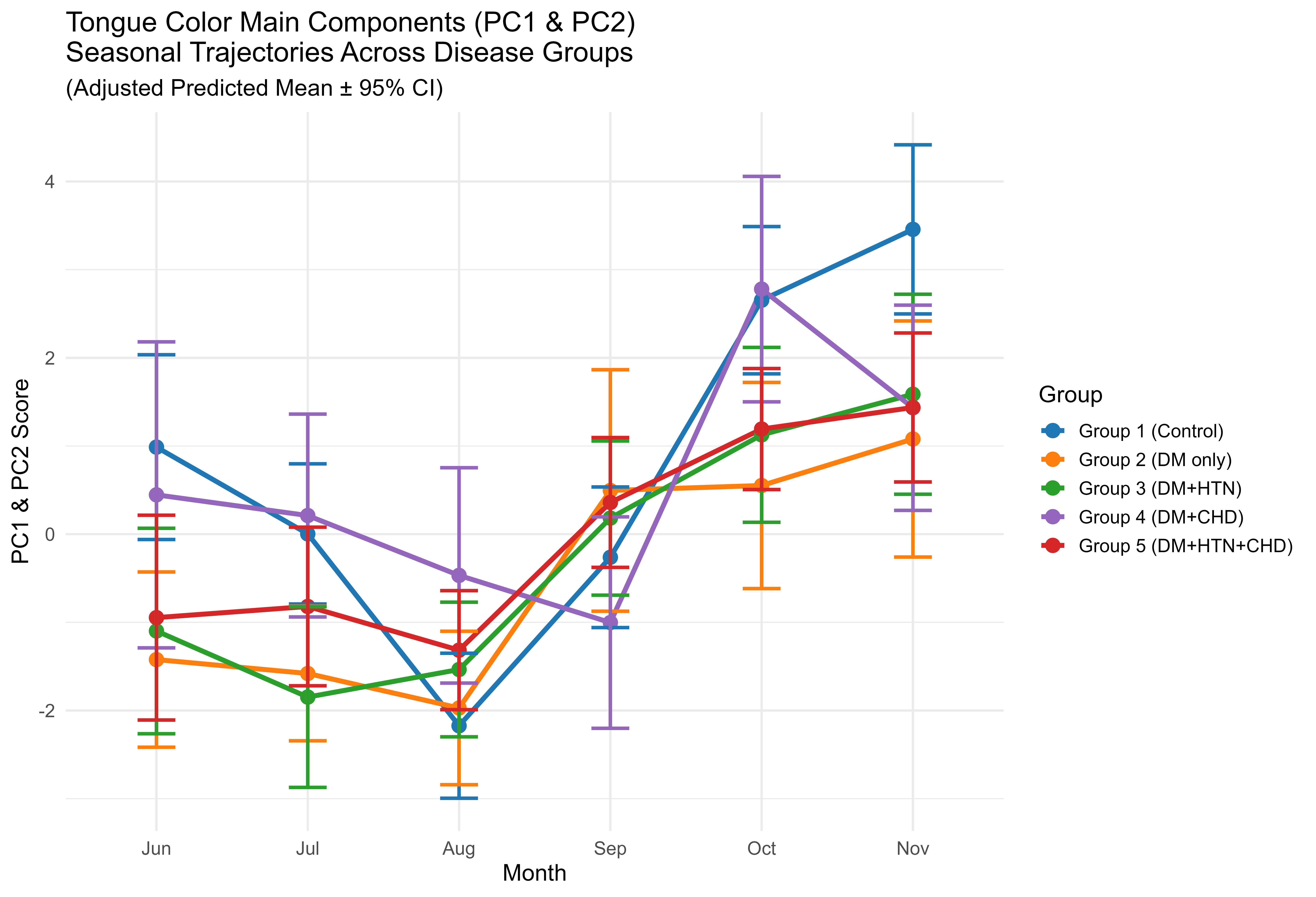

Supplement: Supplementary file 5 [file Image5.png]

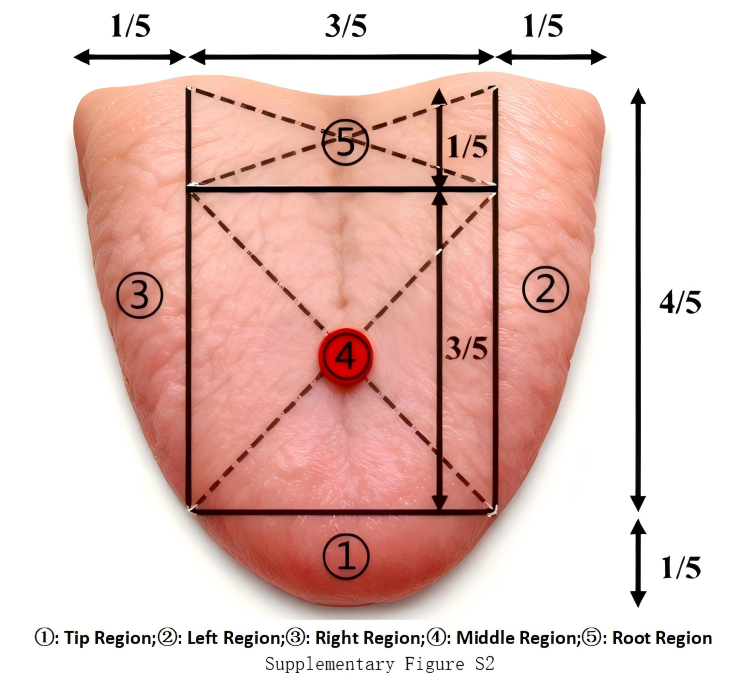

Supplement: Supplementary file 6 [file Image6.png]
